# Antibacterial Activity of Methyl Gallate Isolated from Galla Rhois or Carvacrol Combined with Nalidixic Acid Against Nalidixic Acid Resistant Bacteria 

**DOI:** 10.3390/molecules14051773

**Published:** 2009-05-11

**Authors:** Jang-Gi Choi, Ok-Hwa Kang, Young-Seob Lee, You-Chang Oh, Hee-Sung Chae, Hye-Jin Jang, Dong-Won Shin, Dong-Yeul Kwon

**Affiliations:** 1 Department of Oriental Pharmacy, College of Pharmacy, Wonkwang University, Wonkwang Oriental Medicines Research Institute, Jeonbuk 570-749, Korea; E-mails: jj0038@wku.ac.kr (J-G.C.), kangokhwa@daum.net (O-H.K.), st_andrea81@naver.com (Y-S.L.), ulivuli@daum.net (Y-C.O.), saint197@naver.com (H-S.C.), with-herb@daum.net (H-J.J.); 2 Department of Oriental Medicine Resources, Sunchon National University, Jeonnam 540-742, Korea; E-mail: sdw@sunchon.ac.kr (D-W.S.)

**Keywords:** Nalidixic acid-resistant bacteria, methyl gallate, carvacrol, antibacterial activity, combination

## Abstract

Methyl gallate is a major component of *Galla Rhois*, as carvacrol is of oregano essential oils. Both have shown good antibacterial activity against intestinal bacteria. This study investigated the antibacterial activities of nalidixic acid in combination with methyl gallate and carvacrol against nalidixic acid resistant bacteria. The combined effect of nalidixic acid with methyl gallate and carvacrol was evaluated using the checkerboard method to obtain a fractional inhibitory concentration index. The results showed that the combinations of nalidixic acid + methyl gallate/carvacrol improved nalidixic acid resistant pathogenic bacteria inhibition with synergy or partial synergy activity. Thus, a strong bactericidal effect of the drug combinations was observed. *In vitro* data thus suggested that nalidixic acid combined with methyl gallate and carvacrol may be microbiologically beneficial, rather than antagonists.

## Introduction

The rates of resistance of pathogenic microorganisms to antimicrobial agents are increasing with alarming frequency. The emergence of bacterial resistance to antibiotics has consequently become a worldwide concern [1,2]. Therefore, combination therapy is often beneficial for patients with serious infections caused by drug-resistant pathogens [3,4]. The use of combination therapy can broaden the spectrum of antibacterial activity, minimize the emergence of resistant bacterial variants and can sometimes result in synergic interaction, thereby exhibiting antibacterial activity greater than what would be expected from each drug individually [5]. 

Nalidixic acid is a synthetic antimicrobial drug introduced in 1963 for the therapy of urinary tract infections as it active against the majority of Gram-negative organisms that cause such diseases [6,7]. It is especially active against *E. Coli*, and also often effective against other coli form of bacteria such as *Klebsiella* and *Enterobacter aerogenosa* [6]. Early reports on nalidixic acid indicated the development of single step type resistance, both *in vitro* and *in vivo*. Most recent studies have confirmed that resistance to this drug develops rapidly during treatment, irrespective of the dose used [7], so nalidixic acid resistant pathogenic bacterial infections are becoming more and more common in the clinical setting. To prevent or delay the emergence of resistance, it has been suggested to use nalidixic acid in combination with another antimicrobial agent [7]. 

On the other hand, there has been a considerable interest for the use of plant materials as an alternative method to control pathogenic microorganisms. These plant products have proven to be effective and safe [8]. In this context, carvacrol as a major component of oregano essential oil, exhibits its strong antimicrobial activity [9] by destroying the outer membrane of Gram-negative bacteria, releasing lipopolysaccharides and increasing permeability of cytoplasmic membrane to ATP [10,11]. Other activities include antifungal [12,13] antitoxigenic [14], insecticidal [15], and antiparasitic [16] properties.

Methyl gallate, a major component of *Galla Rhois*, also exhibits strong antimicrobial activity. It is known to possess growth-inhibiting activity against *E. coli*, without adversely affecting the growth of lactic acid-producing bacteria, with the activity being more pronounced by the presence of methyl gallate [17,18]. It is noteworthy that the antibacterial activity of methyl gallate described in this study is in agreement with results reported by others, showing that the activity of *Galla Rhois*, and of other species against bacteria and synergistic combination with ciprofloxacin against *Salmonella* [19], is due to this compound [20,21].

For all these reasons, we decided to study the bactericidal effects of the combinations of nalidixic acid with methyl gallate and carvacrol against nalidixic acid resistant bacteria.

## Results and Discussion

The MICs for methyl gallate and carvacrol against the eight tested strains of nalidixic acid resistant bacteria are shown in Table 1, Table 2. 

**Table 1:** Result of the combined effect of methyl gallate (MG) and nalidixic acid (NA) againstnalidixic acid-resistant pathogenic bacteria.

Strain	Serotypes	^a) ^MIC MG		MIC NA		^b) ^FICI
		Alone (µg/mL)	With NA (µg/mL)	Alone (µg/mL)	With MG (µg/mL)	
CCARM 1	NR-*E.coli*	250	125	250	15.6	0.56
CCARM 2	NR-*E.coli* 078	250	125	250	15.6	0.56
CCARM 3	NR-*S. typhimurium*	500	125	500	31.25	0.31
CCARM 4	NR-*S. derby*	250	125	250	62.5	0.75
CCARM 5	NR-*S. enteritidis*	500	250	500	31.25	0.56
CCARM 6	NR-*S. minesota*	250	500	250	62.5	2.25
CCARM 7	NR-*K. oxytoca*	1000	500	250	15.6	0.56
CCARM 8	NR-*E.cloacae*	250	125	500	31.25	0.56

^a) ^MIC, Minimum inhibitory concentration; ^b) ^FICI, fractional inhibitory concentration index.

**Table 2:** Result of the combined effect of carvacrol (CA) and nalidixic acid (NA) against nalidixic acid-resistant pathogenic bacteria.

Strain	Serotypes	^a) ^MIC CA		MIC NA		^b) ^FICI
		Alone (µg/mL)	With NA (µg/mL)	Alone (µg/mL)	With CA (µg/mL)	
CCARM 1	NR-*E.coli*	250	31.25	250	31.25	0.25
CCARM 2	NR-*E.coli* 078	250	62.5	250	500	2.25
CCARM 3	NR-*S. typhimurium*	250	62.5	500	31.25	0.31
CCARM 4	NR-*S. derby*	250	31.25	250	31.25	0.25
CCARM 5	NR-*S. enteritidis*	500	125	500	15.6	0.28
CCARM 6	NR-*S. minesota*	500	31.25	250	15.6	0.12
CCARM 7	NR-*K. oxytoca*	125	62.5	250	250	1.5
CCARM 8	NR-*E.cloacae*	500	250	500	500	1.5

^a) ^MIC, Minimum inhibitory concentration; ^b) ^FICI, fractional inhibitory concentration index.

The MICs were determined using the broth dilution method. They confirmed the antimicrobial effects we found by the disc diffusion method. Methyl gallate and carvacrol showed antimicrobial activity against all the tested strains. The MICs of methyl gallate against the nalidixic acid resistant bacteria strains ranged from 250 to 1000 µg/mL, and for carvacrol MICs ranged from 125 to 500 µg/mL. 

In order to better assess the efficiency of the natural products, a known antibiotic was used for comparison. The microorganisms used in the experiment are known to our knowledge to be sensitive to norfloxacin (NO) as shown in Table 3. Even though the synergistic activity of methyl gallate + nalidixic acid and carvacrol + nalidixic acid are encouraging, the MIC of norfloxacin appear to be better. This can be explained by the fact that these bacteria are sensitive to norfloxacin but resistant to nalidixic acid. However, the presence of either methyl gallate or carvacrol open the possibility of using nalidixic acid in combination despise the fact that these microorganisms are resistant to it. While further research need to be done, the present combinations validate the no longer prescribed drug and contribute to the fight of drug resistant bacteria.

**Table 3:** Antibacterial activity of nalidixic acid (NA), nalidixic acid (NA) + methyl gallate (MG), nalidixic acid (NA) + carvacrol (CA), and norfloxacin (NO) againstnalidixic acid-resistant pathogenic bacteria (NR).

Strain	Serotypes	^a) ^MIC (µg/mL)			
		NA	NA+MG	NA+CA	^b)^ NO
CCARM 1	NR-*E.coli*	250	15.6	31.25	0.97
CCARM 2	NR-*E.coli* 078	250	15.6	500	0.03
CCARM 3	NR-*S. typhimurium*	500	31.25	31.25	0.97
CCARM 4	NR-*S. derby*	250	62.5	31.25	1.97
CCARM 5	NR-*S. enteritidis*	500	31.25	15.6	1.97
CCARM 6	NR-*S. minesota*	250	62.5	15.6	1.97
CCARM 7	NR-*K. oxytoca*	250	15.6	250	1.97
CCARM 8	NR-*E.cloacae*	500	31.25	500	3.9

^a) ^MIC, Minimum inhibitory concentration; ^b) ^Positive control.

As defined in the checkerboard dilution test protocol, the desired synergy will have a FICI below 0.5. The methyl gallate + nalidixic acid and carvacrol + nalidixic acid combinations exhibited a synergistic effect (1 with methyl gallate + nalidixic acid and 5 with carvacrol + nalidixic acid ) or partial synergy (6 methyl gallate with + nalidixic acid) and indifference (1 with methyl gallate + nalidixic acid and 3 with carvacrol + nalidixic acid) effect (Table 4). 

**Table 4:** ΣFIC values and number of nalidixic acid-resistant pathogenic bacteria showing synergy, partial synergy or indifference with methyl gallate (MG) + nalidixic acid (NA) and carvacrol (CA) + nalidixic acid (NA) combinations.

Combination	^a) ^ΣFICI		No. of strains showing			
	Range	Median	Synergy (%)	Partial Synergy (%)	Additive (%)	Indifference (%)
MG+NA	0.31~2.25	0.76	1	6	0	1
Total			1/8 (12.5)	6/8 (75)	0	1/8 (12.5)
CA+NA	0.12 ~2.25	0.77	5	0	0	3
Total			5/8 (62.5)	0	0	3/8 (37.5)

^a)^ FICI, fractional inhibitory concentration index.

The results thus suggest that the combinations of nalidixic acid + methyl gallate and nalidixic acid + carvacrol exhibited improved inhibition of nalidixic acid resistant bacteria with synergy or partial synergy, as the methyl gallate + nalidixic acid and carvacrol + nalidixic acid combinations inhibited the growth of nalidixic acid resistant bacteria strains at a lower concentration than when the single drugs were assayed separately, thus confirming the hypothesis that antibiotic-resistant inhibitors combined with antibiotics are a potential method for solving the problem caused by resistant bacteria. 

## Experimental

### Plant materials and chemicals

*Galla Rhois*, purchased from the Daehak Hanyak kuk oriental drug store (Iksan, Korea), was authenticated by Dr. D.Y. Kwon. A voucher specimen was deposited in the Laboratory of Herbalogy, College of Pharmacy, Wonkwang University, Iksan, Korea. The EtOH extracts were partitioned with organic solvents of different polarities to yield in sequence *n*-hexane, EtOAc, *n*-BuOH and H_2_O fractions. The EtOAc fraction of each plant was subjected to silica gel chromatography with CHCl_3_-MeOH-H_2_O (lower layers, by volume, 25:7:5, 7:3:1) as the solvents to yield methyl gallate. The structure of the compound was determined by its physico-chemical and spectral data (^1^H-NMR and ^13^C-NMR) which were in agreement with those reported in literature [17,19]. Carvacrol, norfloxacin and nalidixic acid were purchased from Sigma Chemical Co. (St. Louis, MO, USA). 

### Bacterial strains and culture medium

Nalidixic acid resistant bacteria (*E. coli*, *E. coli* 078, *S. typhimurium*, *S. derby*, *S. enteritidis*, *S. minesota*, *K. oxytoca*, *E. cloacae*) were provided by the Culture Collection of Antimicrobial Resistant Microbes (CCARM, Republic of Korea). The bacterial strains were suspended in Mueller-Hinton broth (MHB, Difco, USA) and incubated at 37 °C for 20 h. Muller Hinton agar (MHA, Difco, USA ) was used for the agar diffusion method and MIC.

### Determination of the minimum inhibitory concentrations

The minimal inhibition concentration (MIC) values were determined for the microorganisms we found to be sensitive to methyl gallate and carvacrol during the disc diffusion assay. We prepared the microorganism inocula from 12 h broth cultures and the suspensions were adjusted to a 0.5 McFarland standard turbidity. Susceptibility tests were carried out by the standard broth micro dilution method in accordance with the CLSI guidelines [22] in MHB with an inoculum of approximately 5×10^4 ^CFU/mL. The MHB was supplemented with serial methyl gallate, carvacrol concentrations ranging from 3.9 to 1000 µg/mL, norfloxacin at concentrations from 0.015 to 250 µg/mL, and nalidixic acid at concentrations from 0.97 to 1000 µg/mL. The data were reported as MICs, the lowest concentration of methyl gallate, carvacrol and nalidixic acid inhibiting visible growth after 24 h of incubation at 37 °C [22]. The MICs of nalidixic acid was also determined, and similarly defined as the lowest antibiotic concentration at which no visible bacterial growth was observed.

### The checkerboard dilution test

The antibacterial effects that resulted from combining the two antimicrobial agents were assessed by the checkerboard test. The antimicrobial combination we assayed included methyl gallate plus nalidixic acid and carvacrol plus nalidixic acid. The serial dilutions of the two different agents were mixed in cation-supplemented MHB. The inocula were prepared from colonies that had been grown on MHA overnight. The final bacterial concentration after inoculation was 5×10^4 ^CFU/mL. The MIC was determined after 24 h of incubation at 37 °C. The fractional inhibitory concentration (FIC) index was determined by the formula: FIC index = FIC_A_+ FIC_ B_ = [A]/MIC_ A_ + [B]/MIC_ B_, where [A] is the concentration of drug A, MIC_A_ is its MIC and FIC_A_ is the FIC of drug A for the organism, while [B], MIC_B_, and FIC_B_ are defined in the same fashion for drug B. The FIC index thus obtained was interpreted as follows: <0.5, synergy; 0.5 to 0.75, partial synergy; 0.76 to 1.0, additive effect; >1.0 to 4.0, indifference; and >4.0, antagonism [23]. Finally, the varying rates of synergy between two agents were determined. 

## Conclusions

The *in vitro* activity of methyl gallate and carvacrol against nalidixic acid resistant bacteria and their synergistic, or partially synergistic or indifferent interactions with nalidixic acid were demonstrated for the first time. Methyl gallate and carvacrol have the potential to restore the effectiveness of nalidixic acid against nalidixic acid resistant bacteria, and it could be useful in developing valuable clinical treatments.
